# Methicillin-Resistant *Staphylococcus aureus* (MRSA): Prevalence and Antimicrobial Sensitivity Pattern among Patients—A Multicenter Study in Asmara, Eritrea

**DOI:** 10.1155/2019/8321834

**Published:** 2019-02-06

**Authors:** Eyob Yohaness Garoy, Yacob Berhane Gebreab, Oliver Okoth Achila, Daniel Goitom Tekeste, Robel Kesete, Robel Ghirmay, Ruta Kiflay, Thomas Tesfu

**Affiliations:** Department of Clinical Laboratory Sciences, Asmara College of Health Sciences (ACHS), Asmara, Eritrea

## Abstract

**Background:**

Methicillin-resistant *Staphylococcus aureus* (MRSA) is a well-recognized public health problem throughout the world. The evolution of new genetically distinct community-acquired and livestock-acquired MRSA and extended resistance to other non-*β*-lactams including vancomycin has only amplified the crisis. This paper presents data on the prevalence of MRSA and resistance pattern to other antibiotics on the selected specimen from two referral hospitals in Asmara, Eritrea.

**Method:**

A cross-sectional study was conducted among 130 participants recruited from two referral hospitals in Asmara, Eritrea. Isolation of *S. aureus* was based on culture and biochemical profiles. Standard antimicrobial disks representing multiple drug classes were subsequently set for oxacillin, gentamicin, erythromycin, and vancomycin. Data were analyzed using SPSS version 20 software.

**Results:**

*S. aureus* isolation rate from the 130 samples was 82 (63.1%). Patients <18 years of age were more likely to be colonized by *S. aureus* compared to patients above 61 years. The proportion of MRSA among the isolates was 59 (72%), methicillin-intermediate *S. aureus* (MISA) was 7 (8.5%), and methicillin-sensitive *S. aureus* (MSSA) was 15 (19.5%). The isolates were mostly from the pus specimen in burn, diabetic, and surgical wound patients. Antimicrobial susceptibility test showed that 13 (15.9%) of the isolates were resistant to vancomycin, 9 (11.0%) to erythromycin, and 1 (1.2%) to gentamicin. Coresistance of MRSA isolates to some commonly used antibiotics was also noted: oxacillin/erythromycin 5 (6.1%) and oxacillin/vancomycin 9 (11%). A few isolates were resistant to oxacillin/vancomycin/erythromycin 2 (2.4%) and oxacillin/gentamicin and erythromycin 1 (1.2%).

**Conclusion:**

This study reports a relatively high prevalence of MRSA. Isolates that are resistant to other tested antibiotics including vancomycin are also reported. The data have important implication for quality of patients care in the two settings: antibiotic selection and infection control practices, and the need for additional studies.

## 1. Background

Methicillin-resistant *Staphylococcus aureus* (MRSA) strains or multidrug-resistant *S*. *aureus*, initially described in 1960s, emerged in the last decade as a cause of nosocomial infections responsible for rapidly progressive, potential fatal diseases including life-threatening pneumonia, necrotizing fasciitis, endocarditis, osteomyelitis, severe sepsis, and toxinoses such as toxic shock syndrome [1]. A multifactorial range of independent risk factors for MRSA has been reported in literature and include immunosuppression, hemodialysis, peripheral malperfusion, advanced age, extended in-hospital stays, residency in long-term care facilities (LTCFs), inadequacy of antimicrobial therapy, indwelling devices, insulin-requiring diabetes, and decubitus ulcers, among others [2].

Initially recognized as a problem in healthcare facilities, hence the moniker, healthcare-associated methicillin-resistant *S*. *aureus* (HA-MRSA), new genetically distinct reservoirs of MRSA strains, including community-associated MRSA (CA-MRSA) and livestock-acquired MRSA (LA-MRSA) have been described [3]. Importantly, the rapid evolution of new genetic lineages/clonal complexes (CC) and subtypes and resistance to multiple classes or antibiotics including linezolid (oxazolidinone), the only new class of antibiotic for <20 years, has compounded the public-health crisis associated with MRSA [4, 5].

At present, healthcare-associated methicillin-resistant *S*. *aureus* (HA-MRSA) is associated with significant mortality and morbidity (longer hospital stays) and imposes a serious economic burden on scarce healthcare resources worldwide compared to methicillin-sensitive *S. aureus* (MSSA) [6]. In Europe, data from the European Antimicrobial Resistance Surveillance System (EARSS) reported that prevalence of HA-MRSA in acute care and long-term settings ranged between 1% and 24% with considerable intracountry and intercountry variation [7, 8]. Additional literature from pan-European surveys suggest that MRSA affects >150,000 patients annually in the European Union (EU) and accounts for 380 million Euros in extra in-hospital costs for EU healthcare systems [4] and that the average excess costs per MRSA-positive patient ranged from 5,700 to 10,000 Euros [9].

In the United States (US), a high percentage of S*. aureas* isolates which are the number one cause of nosocomial infections are methicillin resistant [10]. A previous survey, collating national hospitalization, and resistance data spanning 1999–2005 indicated that MRSA-related hospitalizations doubled from 127,036 to 278,203 [11]. Another report also indicated that, in 2005, there were an estimated 94,360 invasive MRSA infections in the US of which 18,000 patients lost their lives [12], a proportion which is higher than HIV/AIDS associated fatalities. These reports should also be interpreted in the context of a meta-analysis which noted that the mortality rate for patients with invasive MRSA infections is significantly higher compared to those with MSSA (odds ratio (OR) 1.93, *p*=0.001) [13].

Although the frequency and impact of MRSA is well documented in western countries, a WHO first global report on antibiotic resistance published in 2014 noted that, in the African region, there exists a major gap in monitoring and tracking antibiotic resistance, with data gathered in only limited number of countries across the continent [14]. Studies indicate that within-country and across-country prevalence of MRSA is heterogeneous with prevalence of 34.6% reported in a setting in Cameroon, [15], 47% (95% CI: 33%–61%) in Ethiopia [16], 3.7% in Kenya [17], 31.5% in Uganda [18], and 9% in Eritrea [19]. Others have commented that available evidence on MRSA in disparate relevant studies yield variable findings; hence, extrapolation of relevant categorical conclusions is difficult [20]. Furthermore, data on a pan-Africa wide scale on the mortality or financial burden of HA-MRSA in the continent are not readily available.

Emphasizing the potential threat MRSA poses to healthcare systems in Africa, the WHO report also noted that, in some parts of Africa, as many as 80% of *S. aureus* infections are resistant to methicillin (MRSA), rendering treatment with standard antibiotics ineffective [14]. In addition, there is a consensus that the emergence of antimicrobial drug resistance (AMR) also threatens life-saving medical technologies such as organ transplants where outcomes are dependent on prevention of surgical site infections.

In Eritrea, limited information exists on prevalence and drug susceptibility patterns of methicillin-resistant *S. aureus* isolated from clinical samples. To the best of our knowledge, this is the second study to report on the prevalence of MRSA in the country. Knowledge of local antimicrobial resistance patterns and of MRSA in particular can be useful to physicians, clinical microbiologists, and public health officials within the country and across the region. The information is also critical for decisions regarding pathogen specific therapy, hospital formulary, and target-oriented infection control policies [21, 22]. The need for the continuous effort directed at monitoring the emergence of resistance by an early detection of strains has also been advised [6].

The main objective of this study was therefore to determine the prevalence of HA-MRSA in patients from selected hospitals in Asmara, Eritrea. An evaluation of the susceptibility patterns of *S. aureus* isolates from the selected specimen to specific antibiotics was also undertaken.

## 2. Materials and Methods

### 2.1. Study Design and Area

Asmara, the capital city of Eritrea, is located in Zoba Maekel, one of the six zones in the country. In this study, two hospitals, Halibet Referral Hospital and Orotta Referral Hospital, were included in the study. The study was conducted at the National Health Laboratory (NHL), which serves as the National Reference Laboratory for Eritrea. Specimens were collected from in-patients with skin and soft tissue infection- (SST-I) purulent discharges of wound and transported (within 2 hours) to the Microbiology Department of National health laboratory (NHL) for processing. Additional information on participants including age, sex, onset of the lesion, previous antibiotic intake, and type and associated medical conditions was also collected. The study was carried out in 2016 (February–May).

### 2.2. Inclusion and Exclusion Criteria

Patients were prescreened by the treating physician for inclusion/exclusion in the study. Inclusion criteria included all inpatients with an active surgical site infection (SSI), infected skin ulcers or burn, skin abscess, diabetic foot ulcers, decubitus ulcers, and infected traumatic would, among others. Exclusion criteria included absence of informed consent.

### 2.3. Sample Collection and Processing

A total of 130 specimens sampled from abscess, burns, surgical wound, and lesions from diabetic patients were collected using sterile swabs. Specimen collection sites were prepared using Levine's technique [23]. Double wound swabs were subsequently taken from each wound. Pus from abscess, purulent discharges of surgical wound, etc. were first inoculated in nutrient agar (NA) and then subcultured in mannitol salt agar (MSA) (Oxoid Limited, UK) and aerobically incubated for 24 hours at 37°C. Blood samples were inoculated on chocolate agar (CA) (Oxoid Limited, UK) and then subcultured in MSA.

Bacterial colonies showing typical characteristics of *S. aureus* including golden yellow color colonies on MSA were subjected to gram staining, catalase test, and DNase test. Mannitol-fermenting, gram-positive bacteria appearing as grape-like clusters and exhibiting catalase positivity were subcultured in DNase agar (Oxoid Limited, UK) and incubated for 24 hrs at 37°C. DNase agar plates were subsequently flooded with HCl (1N) (Oxoid Limited, UK). Isolates exhibiting the ability to hydrolyze DNA were identified as *S. aureus*.

### 2.4. Antimicrobial Sensitivity Testing

A standardized Kirby–Bauer disk diffusion method utilizing the Muller–Hinton Agar (MHA) (Oxoid, Basingstoke, Hampshire, England) plate technique was performed as per the recommendations of the Clinical and Laboratory Standards Institute (CLSI) guidelines [24]. A bacterial suspension equivalent to the 0.5 McFarland turbidity standard was prepared for inoculation. Standard antimicrobial disks representing multiple drug classes were subsequently set for oxacillin (beta lactam) (1 *µ*g), gentamicin (aminoglycosides) (10 *µ*g), erythromycin (macrolides) (15 *µ*g), and vancomycin (glycopeptides) (30 *µ*g). The plates were incubated at 37°C for 24 hours in Mueller–Hinton agar (MHA) supplemented with 2% NaCl. An inhibition zone diameter of each antimicrobial was then measured and interpreted as resistant (R), intermediate (I), and sensitive (S) by comparing with *mecA* negative (*S. aureus* ATCC 29213) and *mecA* positive (ATCC 33591) controls included in each test run. All the antibiotics used were Oxoid, UK products. Inhibition zones were interpreted according to CLSI guidelines [24]. Infections that occurred for more than three calendar days after admission were defined as hospital acquired.

### 2.5. Ethical Approval

Ethical clearance was given by the Ethical Committee of the Ministry of Health (MoH) of the State of Eritrea and Asmara College of Health Sciences (ACHS). The purpose of the study was explained to each of the participants, and informed written consent was subsequently obtained. Parents or legal guardians were required to sign consent forms for underage children (>18 years). Confidentiality of the results was maintained throughout the study period.

### 2.6. Statistical Analysis and Quality Control

The data collected were analyzed by SPSS software statistical application version 20 (SPSS INC, Chicago, IL, USA). The study findings are displayed in tables, charts, cross tabulations. Pearson's Chi-square (*χ*^2^) and Fisher's exact test were used to evaluate the relationship between antimicrobial resistance and specific variables. Logistic regression was used to determine the association between presence of *S. aureus* isolates and nature or source of the clinical specimen. A *p* value <0.05 was considered statistically significant.

During the study period, rigid quality control procedures, as specified by National Health Laboratory (NHL) of the Ministry of Health of the State of Eritrea, were implemented.

## 3. Results

### 3.1. Sociodemographic Characteristics of Participants

A total of 130 patients specimens were collected of which 76 (58.5%) were males and 54 (41.5%) were female. The age range of the study participants was from 3–67. Most of the study participants were between the age groups of 19–40. (Table 1).

### 3.2. Prevalence of *Staphylococcus aureus*

*S. aureus* isolation rate from the 130 samples was found to be 82 (63.1%). Overall, the frequency of isolation in males was 47 (61.8%) and that of females was 35 (64.8%), *p* = value 0.642. The isolation rate of *S. aureus* was significantly associated with the study participants aged below or equal to 18 (AOR, 95% CI: 5.13 (1.50, 17.5), *p*=0.009). However, no significant association was observed with regard to sex (*p*=0.642) (Table 1).

### 3.3. Isolation Rate of *Staphylococcus aureus*

In this study, out of the 103 pus specimens examined, 64 (62.1%) were found to be *S. aureus* positive. Additionally, 9 (60.0%) of the blood specimens were also positive for *S. aureus*. All of the discharge specimens, 12 (100.0%), showed growth for *S. aureus*. It is to be noted here that, the recovery rate of *S. aureus* from all the three specimen types used (i.e. pus, blood and discharge) was significantly associated with a *p* value of 0.005 (Table 2).

### 3.4. Prevalence of MRSA

The prevalence of MRSA was found to be 59(72.0%). The frequency of MRSA in male patients was 55.9% versus 44.1% in female patients. In addition, the 19–40 age group had the highest rate of MRSA isolation with 22(37.3%), followed by ≤18 which had 21(35.6%). Relatively low frequency of MRSA (16.9%) strains were isolated in patients above 61 years of age. Furthermore, frequency of MRSA in patients with abscess, burns, diabetes, and surgical wounds was 15 (62.5%), 12 (60%), 11 (78.6%), and 21 (87.5%) (Table 3).

The major source of MRSA infection was isolated from pus specimens constituting 46 (71.9%) followed by 5 (7.8%) MISA (methicillin-intermediate *S. aureus*). It was followed by discharge of 9 (75.0%) MRSA isolates, 1 (8.3%) MISA isolates, blood 4 (66.7%) MRSA isolates, and 1 (16.7%) MISA isolates (Table 4).

According to our data, the patients with surgical wound had the highest frequency of MRSA (35.6%). Patients with abscess, burns, and diabetics had a frequency of 25.4%, 20.3%, and 18.6%, respectively (Table 5).

### 3.5. Resistance Profile of Organisms

The antimicrobial susceptibility test revealed 13 (15.9%) resistance to vancomycin, 9 (11.0%) to erythromycin, and 1 (1.2%) to gentamicin. Additionally, 6 (7.3%), 4 (4.9%), and 12 (14.6%) isolates exhibited intermediate resistance to vancomycin, gentamicin, and erythromycin, respectively (Table 6).

### 3.6. Multiple Drug Resistance

Among *S. aureus* isolates, 52.4% were resistant to oxacillin only. A small proportion of the isolates were also resistant to a combination of commonly used antibiotics: Oxa/Eryth, 5 (6.1%); Oxa/Gen/Eryth, 1 (1.2%); Oxa/Van, 9 (11%); Oxa/Van/Eryth, 2 (2.4%) (Table 7).

## 4. Discussion

Evolution of methicillin resistance by *S. aureus* has been traced to the acquisition of the exogenous gene (*mec*A) which is part of the staphylococcal cassette chromosome *mec* (SCCmec) (types I–VII) and is under the control of *Mec*I (a repressor) and *Mec*R1 (a transducer) and, when present, the regulatory/signalling proteins of the *blaZ* system [25]. The mecA gene codes for additional penicillin-binding protein (PBP2a), a peptidoglycan transpeptidase, can confer resistance to all *β*-lactam antibiotics (penicillins, cephalosporins, and carbapenems) [25]. Other isolates containing a particular variant of SCCmec types II and III have expanded range of resistance due to the presence of additional resistance genes. The presence of PBP2a, or mecA positivity, can be typed using methicillin or oxacillin (isoxazolyl penicillin), hence the acronym MRSA- or oxacillin-resistant *S. aureus* (ORSA) [26]. This study was designed to determine the occurrence of MRSA in *S*. *aureus* isolates from inpatients at both Orotta Referral Hospital and Halibet Referral Hospital in Asmara, Eritrea. Oxacillin was used as an indicator for MRSA. Erythromycin was also used because it shows high sensitivity (*in vitro*) to MRSA. Vancomycin (a drug of choice for MRSA treatment) and gentamicin were also included for comparison.

According to our data, *S. aureus* isolation rate from the 130 samples was 82 (63.1%). Sex of the respondents was not associated with presence of *S. aureus*. This finding is similar to a recent finding from a study in Ethiopia. [22, 27]. However, isolation rate was significantly higher in patients below 18 years and significantly lower in patients above 61 years. The observed age-related variation in the frequency of *S. aureus* isolation has been reported elsewhere [27, 28]. Admittedly, it must be recognised that the referenced studies used different age groupings; therefore, the comparisons are at the general or trend level. More importantly, factors which contribute to the observed differences remain unclear.

Furthermore, the isolation rate was highest in discharges from wound and abscess (100%), and pus and blood samples had isolation rates of 62.1% and 40.0%, respectively. The higher frequency of *S. aureus* isolation in pus samples compared to blood samples has been reported in other studies in the region and elsewhere [22, 29, 30]. Other investigators have however reported the converse [31]. The observed variation in proportion of *S*. a*ureus* isolates has been attributed to differences in study design and study population [22]. The high prevalence of isolates in surgical wound, diabetic, and burn patients has also been reported [32].

Existing literature on MRSA have demonstrated that there is a significant geographical variation in the frequency of the pathogen within and between countries [20]. In this study, out of the 82 *S. aureus* isolates, 59 (72%) were MRSA, 16 (19.5%) were MSSA, while the remaining 7 (8.5%) were MISA. The reported proportion is significantly higher than previously reported values (9%) from Eritrea [19]. The value was also higher compared to values reported previously in other settings in the region. In sub-Saharan Africa (SSA), several reviews have indicated that the prevalence of MRSA is between 25% and 50% or less than 25% [20, 33]. However, comparatively higher values have been reported in other settings in SSA: Algeria (surgical wounds) (75%) [34]; Egypt (cancer patients) [35]; Nigeria (wound) (73.8%) [36]; Rwanda (multiple sample types) (82%) [37]. High frequency of MRSA has also been reported in other parts of the world: Peru (80%) [38] and in a setting in Colombia (90%) [39].

The intra- and intercountry variation in prevalence of MRSA has been linked to several factors. These include differences in study design, types of the specimen, laboratory procedures, study population, and study duration, among others [16, 22]. For instance, some studies rely entirely on phenotypic procedures for MRSA detection, while others rely on DNA-based techniques (multilocus sequence typing (MLST), whole genome sequencing, microarrays, conventional PCR, and *spa*-typing, among others) for *mecA*—the gold standard. In general, studies relying on genotypic detection by PCR tend to report comparatively lower MRSA prevalence [28]. However, other studies have demonstrated that while the cefoxitin disk diffusion test have good performance, and oxacillin assays have acceptable sensitivity, specificity, positive predictive value (PPV), and negative predictive value (NPV) [26]. Accordingly, we are confident that the comparatively higher values reported in this study are not due to an underperforming diagnostic method, suboptimal quality control during the study, or inadvertent methodological flaws.

How can we then explain the remarkable difference in the prevalence of MRSA between this study and the previous study by Naik et al. [19]? Several factors may be invoked. A notable point is the difference in study settings (private hospital versus public hospitals), sample population (samples from hospital workers and patients versus patients only), and infection control practices in the two settings. Equally likely is the proposition that the data reflect an actual increase in the prevalence of MRSA over the years or a transient local outbreak. The foregoing suggestion, along with issues highlighted earlier, e.g. study design, should draw our attention to the possibility that the data might overestimate the actual prevalence of MRSA in these settings and that results should not be generalised. The latter proviso is important since there is a tendency by some reviewers to generalise the result of a single-center study within a country to the entire country.

On the either way, the finding should raise concern particularly when viewed in light of the fact that these findings contradict the previous conclusion by Naik and Teclu that the spread of MRSA in community and hospital settings in Eritrea is limited [19]. Add to this, the commentary by Falagas et al. is that the observed low prevalence might be attributed to underutilization of antibiotics in Eritrea leading to lower selection pressure for MRSA [20]. In contrast, our findings appear to partially complement recent reports highlighting high frequency of community associated (CA) Panton–Valentine leucocidin- (PVL-) positive MRSA among recently arrived Eritreans (with common skin infections) to Europe [40, 41]. Underlying this report was the conclusion that the frequency of MRSA must be high in Eritrea. Reinforcing the same point, Jaton et al. emphasised that their results, which uncovered a clustering of closely related CC15 and CC152 PVL—producing MRSA strains in the same group, denoted the probable high frequency of these strains in Eastern Africa [42].

The high prevalence of MRSA in two major referral hospitals in the country should raise concern over possible interhospital transfer of colonized patients. Dissemination of MRSA in a country via interhospital transfer of colonised patients is a well-documented phenomenon. A study applying mathematical modelling to national data on patterns of patient referral [43] and the first comprehensive molecular characterization undertaken in South African, Kwazulu Natal province, which profiled isolates from 14 hospitals, noted this to be the case [21]. In fact, interhospital transfer of patients from regional health facilities to national public referral hospitals is a common practice in SSA, Eritrea included. The idea that these facilities can act as significant reservoirs of infection has implication of MRSA control strategies in the country. Another concerning finding relates to the nature of the patients sampled: surgical wound, burn, and diabetic mellitus (DM) patients. Often, these patients have extended stays in general in-patient wards are at an increased risk of invasive bacterial diseases; hence; possible colonization by MRSA and implication on dissemination dynamics should be a foremost concern.

Data on the overall antimicrobial resistance (AMR) to other antibiotics were as follows: 15.9% of the isolates were resistant to vancomycin, 1.2% to gentamicin, and 11% to erythromycin. In addition, 13.1% of the isolates were resistant to both oxacillin and vancomycin, 3.3% of the isolates were resistant to oxacillin, vancomycin, and erythromycin, and 1.64% isolates were resistant to oxacillin, gentamicin, and erythromycin. Isolates with these profiles have been isolated in the region [22]. The pattern of multiclass antibiotic resistance reported in this study is in concordance with studies from other settings in SSA [44]. The high frequency of multiple drug resistant- (MDR-) *S. aureus* isolates in the region has been linked to improper use of antibiotics, including in animal husbandry, self-medication, and substandard infection control and prevention practices [20, 44]. On the contrary, the possible spread of some of the isolates identified in this study also has public health implications.

Interestingly, higher sensitivity pattern was observed for gentamicin (93.9%) compared to vancomycin (76.8%). High susceptibility to gentamicin has also been reported by several authors working in SSA [17, 21, 22, 45]. However, other authors have reported considerably higher frequency of gentamicin and erythromycin resistance [46]. The explanation for the observed variability may be linked to the differences in antibiotic prescription practices between countries in SSA.

Like other studies conducted in the region [22, 46], this study confirms the presence of vancomycin-resistant *S. aureus* (VRSA) in the country—it should be noted that the previous study by Naik et al. did not provide data on *S. aureus* susceptibility tests to vancomycin. The recovery of *S. aureus* isolates that are resistant to both vancomycin and oxacillin also parallels reports from recent studies in the region [22, 32, 33, 46]. In contrast, some studies in SSA reported 100% susceptibility to vancomycin [17, 21, 44]. Falagas et al. estimated that the susceptibility of MRSA isolated in Africa to VRSA is between 82 and 100% [20]. These estimates and the findings of this study contradict a recent conclusion by Kong et al. that VRSA strains are rare and that there is limited evidence of increasing frequency [6].

Although the epidemiology of VRSA is poorly documented in SSA, it appears to be heterogeneous with important regional variation-zero isolates in Southern Africa and a substantial presence in Northern and some parts of Eastern Africa. As for vancomycin as the treatment of choice for MRSA [17], it is our position that the substantial proportion of vancomycin-resistant MRSA and vancomycin-intermediate traits reported in this study and elsewhere in the region presupposes the need for locally appropriate prescription recommendations based on alternative antibiotics or combination of existing low cost antibiotics. Furthermore, *in vitro* susceptibility testing of MRSA isolates against vancomycin or suitable combinations should be undertaken before commencement of therapy.

## 5. Limitation

Although this study presents data from a setting where information on AMR is extremely limited, we have to acknowledge some limitations. The generalizability of the data might be compromised by sampling biases—the true case load for these hospitals is still unknown. In addition, the data should not be generalised to the entire country. Furthermore, the distinction between HA and MRSA and CA and MRSA is blurred. Therefore, the actual source of infection is uncertain. In addition, we did not collect data on a range of vital parameters including duration of stay in the hospital, region of residence, history of antimicrobial use, and exposure to livestock, among others. Molecular characterisation of isolates and associated virulence factors were also not undertaken. In spite of the highlighted limitations, it is still our position that these studies present vital information on the burden of MRSA and associated AMR patterns. Therefore, the data have important implication for quality of patients care (high frequency of MRSA is a marker of poor patient care), empirical antibiotic selection, infection control practices, and the need for continuous AMR monitoring, among others.

## 6. Conclusion

Antibiotic resistance is a growing public health crisis in SSA, and the urgent need for local studies has been proposed. However, the epidemiology of AMR in the region is poorly understood. This situation applies to MRSA. In this study, we presented data on the frequency of MRSA in two referral hospitals in Asmara, the capital city of Eritrea. In contrast to a previous study, the present study found alarming levels of MRSA isolates (72%) and high cross-resistance of the isolates to other antibiotics (Vancomycin in particular). This denotes a substantial increase after 2009. The highlighted increase accentuates the need for a comprehensive drug resistance surveillance and containment system. A comprehensive tracking system should be able to capture data on emerging AMR trends, report infections from different healthcare sectors (acute, long-term, ambulatory) and veterinary care across the country, and recognise high risk patients, among others. This information can subsequently be leveraged to design good infection control practices and optimal usage of antimicrobial agents in the country. We recognise that these propositions can only be implemented in the long term. Currently, reevaluation of existing infection control practices, implementation of more effective practices (screening of MRSA carriers, isolation or cohorting of patients, colonised healthcare workers, and environmental decontamination, among others) should suffice. Investment in laboratory infrastructure and allied personnel should also be prioritised.

## Figures and Tables

**Table 1:** Association of *S. aureus* among participants with regard to age, gender, and case.

Variable		Presence of *S. aureus*		COR (95% CI)	*p* Value	AOR (95% CI)	*p* Value
		No (*N*(%))	Yes (*N*(%))				
Sex	Female	19 (35.2)	35 (64.8)	1.12 (0.51, 2.45)	0.78	-	-
	Male	29 (38.2)	47 (61.8)	1		-	-

Age group	<12	3 (15)	17 (85.0)	5.67 (1.23, 26.13)	0.26	6.23 (1.4, 27.8)	0.017
	13–18	3 (21.4)	11 (78.6)	3.6 (0.72, 17.42)	0.12	4.03 (0.87, 18.8)	0.075
	19–40	17 (34)	33 (66)	1.72 (0.56, 5.27)	0.343	2.13 (0.76, 6.02)	0.152
	41–60	14 (56)	11 (44)	0.88 (0.27, 2.81)	0.637	0.75 (0.22, 2.5)	0.806
	≥61	11 (52.4)	10 (47.6)	1	0.12	1	0.028

Case	Abscess	10 (21)	24 (29)	1.6(0.56, 4.53)	0.38		
	Burn	9 (19)	20 (24)	1.09 (0.37, 3.19)	1.09		
	Diabetic	15 (31)	14 (17)	0.66 (0.22, 1.96)	0.451		
	Surgical wound	14 (29)	24 (29)	1			

COR: crude odds ratio; AOR, adjusted odds ratio; CI, confidence interval.

**Table 2:** Isolation rate of *S. aureus* from clinical specimens.

Specimen type	Presence of *S. aureus*		Total (*N*)	*p* Value
	Yes (*N*(%))	No (*N*(%))		
Pus	64 (62.1)	39 (37.9)	103	0.005
Blood	6 (40.0)	9 (60.0)	15	
Discharge	12 (100.0)	0 (0.0)	12	

**Table 3:** Association of methicillin-resistant pattern of *S. aureus* with regard to age and gender among study participants.

Variable		MRSA (*N*(%))	MISA (*N*(%))	MSSA (*N*(%))	*S. aureus* (*N*)
Sex	Female	26 (44.1)	2 (28.6)	7 (43.8)	35
	Male	33(55.9)	5 (71.4)	9 (56.2)	47

Age group	≤18	21 (35.6)	2 (28.6)	5 (31.2)	28
	19–40	22 (37.3)	3 (42.69)	8 (52.0)	33
	41–60	6 (10.2)	2 (28.6)	3 (18.8)	11
	≥61	10 (16.9)	0 (0.0)	0 (0.0)	10

Case	Abscess	15 (62.5)	3 (12.5)	6 (25)	24
	Burn	12 (60)	3 (15)	5 (25)	20
	Diabetic	11 (78.6)	1 (7.1)	2 (14.3)	14
	Surgical wound	21 (87.5)	0 (0)	3 (12.5)	24

MRSA: methicillin-resistant *S. aureus*; MISA: methicillin-intermediate *S. aureus*; MSSA: methicillin-sensitive *S. aureus*.

**Table 4:** Methicillin-resistant *S. aureus* association with different types of clinical specimens.

Specimen type	MRSA (*N*(%))	MISA (*N*(%))	MSSA (*N*(%))	Total (*N*)
Pus	46 (71.9)	5 (7.8)	13 (13)	64
Blood	4 (66.7)	1 (16.7)	1 (16.7)	6
Discharge	9 (75.0)	1 (8.3)	2 (16.7)	12
Total	59 (72.0)	7 (8.5)	16 (19.5)	82

**Table 5:** Frequency of MRSA in different types of clinical samples and cases.

MRSA				
Type of case	Pus (*N*(%))	Blood (*N*(%))	Discharge (*N*(%))	Total (*N*)
Burn	12 (100)	0 (0)	0 (0)	12 (20.3)
Diabetic	9 (81.8)	2 (18.2)	0 (0)	11 (18.6)
Surgical wound	18(85.7)	2 (9.5)	1 (4.8)	21 (35.6)
Abscess	7 (46.7)	0 (0)	8 (53.3)	15 (25.4)
Total	46 (78)	4 (6.8)	9 (15.3)	59 (100)

**Table 6:** Antimicrobial sensitivity pattern of *S. aureus* strains to different antimicrobial agents.

Antibiotics	Resistant (%)	Intermediate (%)	Sensitive (%)
Vancomycin	13 (15.9)	6 (7.3)	63 (76.8)
Gentamicin	1 (1.2)	4 (4.9)	77 (93.9)
Erythromycin	9 (11.0)	12 (14.6)	61 (74.4)
Methicillin	59 (72.0)	7 (8.5)	16 (19.5)

**Table 7:** Multidrug resistance nature of *S. aureus* isolates.

Antimicrobial	Number resistant (*N*)	Resistant (%)
Eryth	1	1.2
Oxa	43	52.4
Oxa + Eryth	5	6.1
Oxa + Gen + Eryth	1	1.2
Oxa + Van	9	11.0
Oxa + Van + Eryth	2	2.4
Sensitive	19	23.2
Van	2	2.4
Total	82	100.0

Oxa: oxacillin; Eryth: erythromycin; Gen: gentamicin; Van: vancomycin.

## Data Availability

The data used to support the findings of this study are available from the corresponding author upon request.

## Abbreviations

AMR: Antimicrobial resistance
SCCmec: Staphylococcal cassette chromosome mec
CC: Clonal complex
PVL: Panton–Valentine leukocidin
MIC: Minimum inhibitory concentration
SSI: Skin and soft tissue infection
MSSA: Methicillin-susceptible *Staphylococcus aureus*
MRSA: Methicillin-resistant *Staphylococcus aureus*
LTCFs: Long-term care facilities
HA-MRSA: Hospital-associated MRSA
CA-MRSA: Community Associated MRSA
LA-MRSA: Livestock-acquired MRSA
EARSS: European antimicrobial resistance surveillance system
EU: European Union
CLSI: Clinical and Laboratory Standard Institute
MDR: Multidrug resistance
MHA: Muller–Hinton agar
NHL: National Health Laboratory
AOR: Adjusted odds ratio
MISA: Methicillin-intermediate *S. aureus*
PBP2a: Penicillin-binding protein
ORSA: Oxacillin-resistant *Staphylococcus*
VRSA: Vancomycin-resistant *Staphylococcus aureus*
MLST: Multilocus sequence typing
PCR: Polymerase chain reaction.
